# Determinants of SARS-CoV-2 infection across three sentinels sites in Benin during 2021: A multicentric surveillance study

**DOI:** 10.1371/journal.pgph.0004227

**Published:** 2025-02-13

**Authors:** Aurore Atchade, Anges Yadouleton, Marc Fiogbe, Daleb Abdoulaye Alfa, Emmanuel Yovo, Jean-Yves Le Hesran, Sandrine Hounsa, Cédric Bationo, Antía Figueroa-Romero, Jean Gaudart, Raquel González, Emmanuel Bonnet, Achille Massougbodji, Gilles Cottrell

**Affiliations:** 1 Institut de Recherche Clinique du Bénin - IRCB, Abomey-Calavi, Bénin; 2 Ministère de la Santé du Bénin, Laboratoire des Fièvres Hémorragiques Virales du Bénin, Cotonou, Bénin; 3 Université Paris Cité, IRD, MERIT, Paris, France; 4 Aix Marseille Univ, IRD, INSERM, SESSTIM, ISSPAM, UMR1252, APHM, Hop Timone, BioSTIC, Biostatistic and ICT, Marseille, France; 5 Barcelona Institute for Global Health (ISGlobal), Hospital Clínic- Universitat de Barcelona, Barcelona, Spain; 6 Manhiça Health Research Center (CISM), Manhica, Mozambique; 7 Université Paris 1 Panthéon-Sorbonne, IRD PRODIG UMR 215, CNRS AgroParisTech 5, cours des Humanités, Aubervilliers, France; NIE: National Institute of Epidemiology, INDIA

## Abstract

In the context of the COVID-19 pandemic, Benin, like other regions in sub-Saharan Africa and around the world, has been grappling with a public health crisis since march 2020. The country responded to this crisis by implementing adaptive response measures. The STREESCO project is part of this dynamic effort, strengthening epidemiological surveillance at three sentinel sites. This study thoroughly examines the determinants of SARS-CoV-2 infection. This involved strategic support based in Cotonou, Allada, and Natitingou. Data collection took place from march 1 to november 30, with individuals voluntarily undergoing COVID-19 screening at dedicated health units. Before participation, free and informed consent was obtained. The collected data included sociodemographic information, clinical details, and the results of COVID-19 tests. A multivariate logistic regression model was used to identify factors associated with SARS-CoV-2 infection. Surveillance included 4178 participants, with a male/female ratio of 0.98 and a median age of 33 (IQR: 25-45). Across all sites, 13.12% of participants tested positive for SARS-CoV-2, and 85.40% of them had a SARS-CoV-2 infection.of participants were symptomatic at baseline. The proportion of positivity was 6%, 11% and 28% respectively during phases 1, 2 and 3 of the national strategy. Identified risk factors included the Allada site (aOR 2.04, 95%CI 1.59-2.62), phase 3 (aOR 3.16, 95%CI 2.34-4.27), phase 2 (aOR 1.67, 95%CI 1.12-2.51), secondary (aOR 1.67, 95%CI 1.12-2.51) and higher level of education (aOR 1.83, 95%CI 1.22-2.74), respiratory symptoms (aOR 1.88, 95%CI 1.40-2.53), and/or anosmia/ageusia (aOR 1.88, 95%CI 1.48-2.38). Conversely, the Natitingou site (aOR = 0.29, 95%CI: 0.20-0.42), group living (aOR = 0.75, 95%CI: 0.60-0.94), and digestive symptoms (aOR = 0.70, 95%CI: 0.54-0.91) were associated with a reduced risk of infection. These results underscore the importance of active surveillance during crisis situations, ensuring the acquisition of reliable and persuasive data to enhance individual care and guide health policies.

**Trial registration:**

NCT06170320 (retrospectively registered on December 21, 2023).

## Introduction

In may 2023, the World Health Organisation (WHO) lifted the global health emergency caused by COVID-19 [1]. In total, there were more than 650 million cases of coronavirus and six million deaths worldwide [2]. In Africa, the number of cases and deaths relative to the population appears to be lower than elsewhere in the world [3]. Nevertheless, numerous seroprevalence studies have shown that the virus has circulated widely in the African population [3–6]. The incidences reported by national statistics therefore probably underestimated the true incidence of the disease in a population of 11 million inhabitants [7,8].

In Benin, during the three years of the health crisis, four epidemic waves due to COVID-19 were observed, with almost 28,000 positive cases and 163 deaths reported [9]. A response to the pandemic was rapidly put in place following the detection of the first positive case in march 2020. The authorities quickly implemented preventive health measures based on a system for triaging patients, screening and treating travellers arriving or leaving Benin by air. In addition to the barrier measures recommended by the WHO, such as hand hygiene, wearing masks, physical distancing, self-isolation, case detection and quarantine, a cordon sanitaire was put in place between march and may 2020 to curb the spread of the disease from the south of the country where the first cases were detected [10–12].

During the pandemic, increasingly targeted response measures were implemented [12]. Thus, from june 2020 to may 2021, Benin moved from a strategy of mass screening of volunteers regardless of their clinical status, to a strategy of routine screening of symptomatic suspect cases. Public health facilities have been empowered to screen for COVID-19, in particular through the use of Rapid Diagnostic Tests (RDT) in 2021, followed by vaccination against COVID-19 [10,12]. Worldwide and in Africa, studies carried out in 2020 showed that characteristics linked to exposure (travel, trade, work-related travel), socio-demographics (age, sex, race, profession), environment (region, climate, community life, type of housing) and clinical features (fever, headache, cough, sore throat, anosmia, ageusia) of patients screened could be predictive of SARS-CoV-2 infection. Aspects such as urban density (urban/rural area), urban population and the existence of conflicts were also found to be factors in the spread of infection [13–17]. These characteristics clearly define specific groups at greater risk of infection. In order to better guide COVID-19 response strategies, it is therefore vital to identify and target these groups in our actions.

In 2021, the STREESCO (STREngthening Epidemiological Surveillance of COVID-19) project was initiated in Benin to support the beninese government in its response. The project set up a sentinel surveillance of the COVID-19 pandemic at three different sites in the country in Cotonou (the economic capital), Allada (a semi-rural town about 50kms north of Cotonou) and Natitingou (a city in the north of the country close to the border with Burkina Faso). We report here the analyses of data collected during epidemiological field surveillance with COVID-19 virological tests in Benin in order to analyse the potential environmental, socio-demographic and clinical determinants of SARS-CoV2 infection in the project sentinel sites.

## Methods

### Ethical considerations

This study received approval from the relevant authorities, notably the Benin Ministry of Health (MS authorisation reference: N°0126-2021/MS/DC/SGM/SP), the Benin National Institute of Statistics and Demography (INStaD statistical visa reference: N°27/2020/MPD/INSAE/DCSFR), and the Benin National Health Research Ethics Committee (CNERS ethical authorisation reference: N°129/MS/DFRMT/CNERS/SA). The investigators then approached potential participants to present the objectives of the study. It was emphasized that participation was voluntary, and those who chose to take part gave their signed informed consent. For participants under 18 years of age, written consent was obtained from parents or legal guardians in addition to the assent of participants over the age of 10. Each participant received a copy of the information form and signed consent form.

### Study design

**Fig 1:**
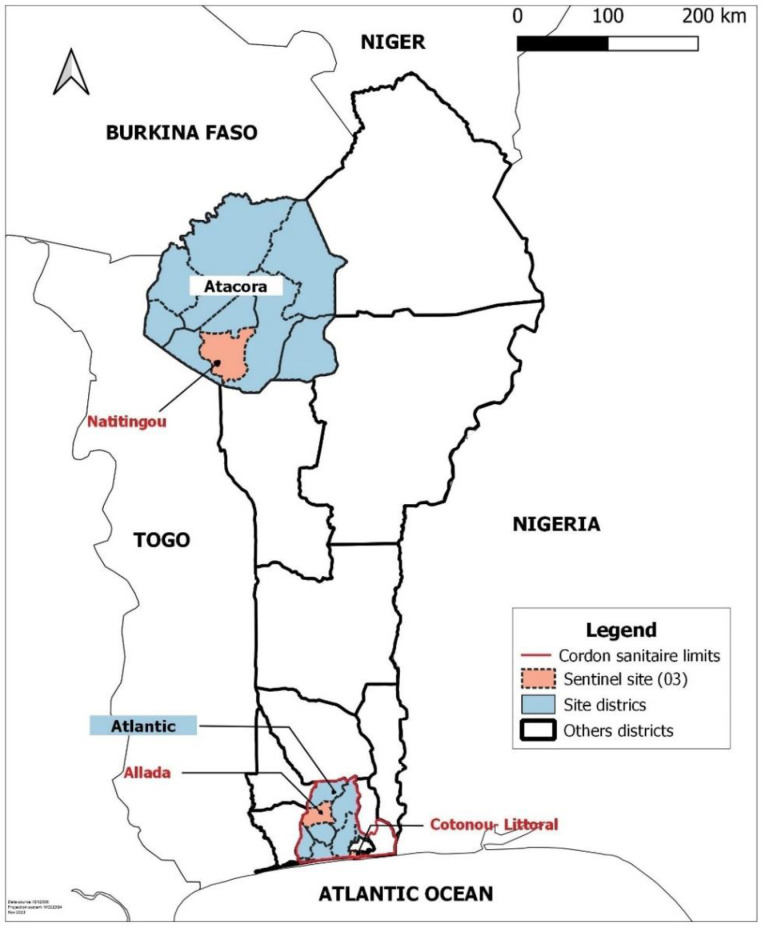
Sentinel study sites by district, Benin 2021.

**Fig 2:**
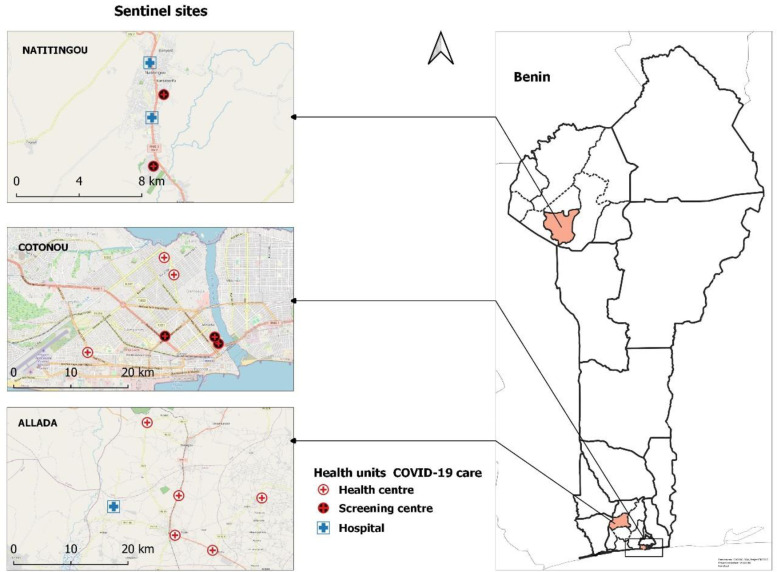
Location of the centres involved in the response to COVID-19 at the sentinel sites of study in Benin.

The study sites were Cotonou, the economic capital with a population of 679,012 according to the RGPH4-2013 census, Allada, a town on the border of the cordon sanitaire with 127,512 inhabitants [11] and Natitingou, a town in northern Benin outside the cordon sanitaire, close to the border with Burkina Faso, with 103,843 inhabitants according to the same census [18] (see Fig 1 URL: https://public.opendatasoft.com/explore/dataset/world-administrative-boundaries/export/). This study was conducted in sixteen (16) COVID-19 screening centers, health centers, and public hospitals in Benin, in collaboration with the Ministry of Health. These centers were selected based on their attendance and the category of people they serve. The study focused on the evolution of the pandemic in the population, and these centers were places where active cases of the disease without severe symptoms requiring hospitalization were more likely to be found. It was a cross-sectional study with prospective data collection that took place from March 1 to November 30, 2021. Surveillance included people who presented themselves spontaneously at these centers for having been in contact with a suspected COVID-19 case (at home, work, market, or during other daily activities) or for having traveled nationally or internationally in the past two weeks. (see Fig 2; URL: https://public.opendatasoft.com/explore/dataset/world-administrative-boundaries/export/). The map of the study sites has been built using the free version of the QGIS 3.22 software [19].

The study was closely linked to the national strategy in place. The response to COVID-19 in Benin has been adjusted on several occasions to reflect the dynamics of the COVID-19 pandemic and the availability of diagnostic tools in the country [12]. As a result, the study period can be divided into 03 phases marked by changes in the screening site, the targeted population and the biological tests (Table 1).

**Table 1:** Different phases of the national response strategy to COVID-19 in Benin between March and November 2021.

	Phase 1	Phase 2	Phase 3
**Period**	01 march 2021-31 may2021	01 june 2021-15 august 2021	15 august 2021-30 november 2021
**Screening location**	COVID-19 screening centres	Health centres and public hospitals	Health centres and public hospitals
**Targeted population**	Asymptomatic and symptomatic subjects	Symptomatic subjects	Symptomatic subjects
**Biological test performed**	RT-PCR COVID-19	RT-PCR COVID-19	- RT-PCR COVID-19 - COVID-19 antigenic RDT

### Data collection

A questionnaire was administered to every screening volunteer or patient recommended for a COVID-19 biological test who attended one of the study sites. This made it possible to collect socio-demographic (sex, age, etc.), anthropometric (weight, height, body mass index, etc.), clinical (presence of symptoms) and biological data (COVID-19 diagnosis). Participants were seen by a doctor, who carried out the clinical examination and the COVID-19 biological test.

The clinical data collected were grouped into five categories. Functional symptoms included fatigue/asthenia, muscle pain, anorexia, headache, muscle soreness; general symptoms included fever with a temperature ≥ 37.5°C, chills and weight loss. Respiratory symptoms included cough, nasal discharge, bleeding or obstruction, and respiratory distress. Digestive symptoms included nausea, vomiting, constipation, diarrhea, abdominal pain and abdominal bloating. Specific symptoms included anosmia and/or ageusia. The data was collected by the same field team of 6 investigators in the health centres using a standardised questionnaire. The data collected were completely anonymized and stored on a SurveyCTO platform server secured by a user name and password.

### Laboratory procedure

During the study, two tests were used to determine the virological status of participants: RT-PCR followed by COVID-19 antigenic RDTs (Table 1). These tests were performed on naso-paharyngeal samples for subjects over 14 years of age, and on oro-pharyngeal samples for children under 14. Initially, selected respiratory samples were analyzed by RT-PCR, a real-time reverse transcription test using primers targeting regions of the viral genome. The primers used for the RT-PCR were the COVID-19 kits from TIB Molbiol. The PCR analyses were carried out at all the sentinel sites in laboratories associated with the study sites under the supervision of the national viral haemorrhagic fever laboratory, which was fully equipped to maintain cold chains. Test turnaround time was approximately 24 hours at the time of examination. COVID-19 antigenic RDTs detected specific antigens (proteins) produced by the virus [20,21]. The RDTs used were the Panbio™ COVID-19 Ag Rapid Test Device from Abbott. RDT tests were carried out on site, and results obtained within 15 minutes. In the event of a positive test result, the participant was declared a confirmed COVID-19 case, and treated in accordance with Benin’s national treatment guidelines [22,23]. These kits and RDTs were the same used throughout the study period for all samples at the 03 study sites.

### Statistical analysis

To report the results of the questionnaire, the datasets were downloaded from the SurveyCTO server and imported into Stata version 14.2 (Stata Corp, College Station, TX) for statistical analyzes. Descriptive statistics were generated. Categorical variables were described by absolute/relative frequencies and their 95% confidence intervals. Quantitative variables were described by their median and interquartile range. The chi-squared trend test was used to compare categorical variables. The Mann-Whitney U non-parametric test was used to compare medians between participants not infected with SARS-CoV-2 and those positive after the test. A p-value < 5% was considered significant.

The outcome variable of the study was the participants’ infection status (SARS-CoV-2 infection positive or negative). Factors associated with SARS-CoV-2 infection by study site, by phase of strategy change, and by type of biological test performed were identified using univariate and multivariate logistic regression models.

In the multivariate model, all variables presenting a p-value < 0.2 in univariate analysis and/or an effect size such as |1-OR| > 0.5 were retained. Additionally, all clinical factors (variables of major interest) were forced into the multivariate model. A backward step was then carried out to retain only variables with a value of p < 0.05 p-value <5% and/or an effect size such as |1-OR| > 0.5.

## Results

**Fig 3:**
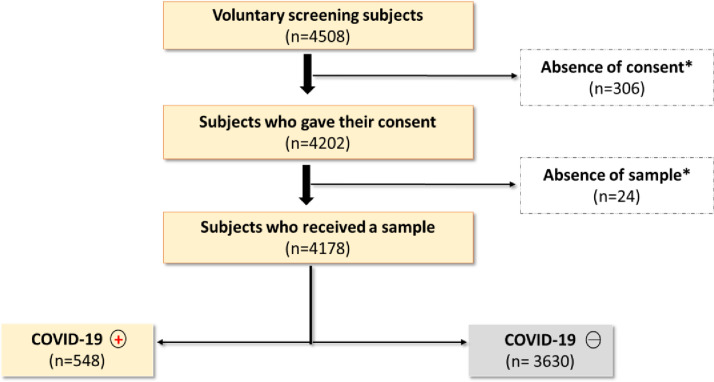
Flowchart diagram of the study.

The flowchart (see Fig 3) represents the process of selecting participants for the study. Approximately 4,500 participants were approached at all sentinel sites. After identification, approximately 7% refused to participate in the study and less than 1% of those who agreed to participate were unable to provide a sample for the COVID-19 test.

A total of 4178 participants were included at the three sites over the study period, 52.37% of whom were recruited in Cotonou. The sex ratio (M/F) was 0.98. The median age was 33 years [IQ: 25-45] and 80% of participants aged between 18 and 60 years. Of the study participants, 37% were employed in the public or private sector. Also 86% of the participants were educated, with an over-representation of those with a high level of education (38%).

In addition, around 64% of participants presented with symptoms at inclusion over the entire study period, whereas the proportion of symptomatic subjects was 34% during phase 1, when screening was open to both symptomatic and asymptomatic subjects (Table 2).

**Table 2:** Epidemiological and clinical profile of volunteers for COVID-19 screening in Cotonou, Allada and Natitingou in 2021.

Characteristics	*n*	% or median (IQR)or mean^*^ *(± SD)*
**Residence area**		
Cotonou	2188	52.37
Allada	1036	24.80
Natitingou	954	22.83
**Phase**		
Phase 1	2026	48.50
Phase 2	964	23.07
Phase 3	1188	28.43
**Gender**		
Male	2167	50.53
Female	2111	49.47
**Age (year)** All participants	4178	33 y (IQR, 25-45)
[01 month–10 y]	270	6.46
[10–18 y]	121	2.90
[18–35 y]	1802	43.13
[35–60 y]	1614	38.63
≥60 y	371	8.88
**BMI (kg/m**^**2**^)	3064	24.22 (IQR, 21 .61-27.47)
<18.5	188	6.14
[18.5–25]	1520	49.61
[25–30]	942	30.74
≥30	414	13.51
**Level of education**		
Out of school	579	13.86
Primary	800	19.15
Secondary	1221	29.22
Higher	1578	37.77
**Nationality**		
Beninee	3981	95.28
Foreign	197	4.72
**Marital status**		
Single	1257	31.97
Married	2675	68.03
**Profession**		
School pupil/student	537	12.85
Farmer	231	5.53
Worker	197	4.72
Craftsman	350	8.38
Shopkeeper/retailer	504	12.06
**Employee**	1535	36.74
Other^a^	213	5.10
No professional activity	611	14.62
**Type of habitat**		
Individual	2733	65.41
Group	1445	34.59
**Clinical status**		
Symptomatic	2668	63.86
Asymptomatic	1510	36.14
**Self-medication with chloroquine**		
Yes	1468	55.02
**Diabetes**		
Yes	94	2.25
**High blood pressure**		
Yes	446	10.67
**Chronic lung disease**		
Yes	313	7.49
**Chronic liver disease**		
Yes	20	0.48
**Functional symptoms**		
Present	2443	58.47
**General symptoms**		
Present	1482	35.47
**Respiratory symptoms**		
Present	2318	55.48
**Anosmia/ageusia**		
Present	678	16.23
**Digestive symptoms**		
Present	756	18.09

^a^Other Profession: nun, priest, tradipractician.

Abbreviations: IQR, interquartile range; SD, standard deviation; BMI, Body Mass Index.

Of the 4178 COVID-19 biological tests performed, 72.36% of these tests were RT-PCR COVID-19. The proportion of positive cases of SARS-CoV-2 infection in our study was 13.12%. Precisely, 9.38% of COVID-19 RT-PCRs were positive and 25.49% of COVID-19 antigenic RDTs were positive.

**Fig 4:**
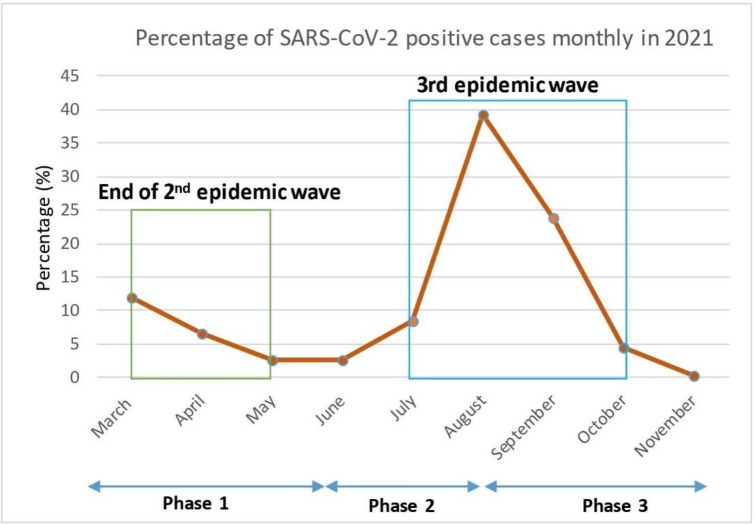
Cases testing positive for SARS-CoV-2 infection between March and November 2021 in Cotonou, Allada and Natitingou.

Of the four waves of COVID-19 epidemics recorded in Benin during the pandemic, the STREESCO study was set up at the end of the 2nd epidemic wave and fully covered the 3rd wave. From March to June 2021, a progressive decrease in the COVID-19 incidence rate was clearly observed from the start of the study to May 2021. In July 2021, the 3rd epidemic wave began, reaching a peak of 215 positive cases in August 2021 (see Fig 4).

### Determinants of SARS-CoV2 infection

In multivariate logistic regression analysis, participants from the Allada site, those with a higher level of education, including high school, and those with respiratory symptoms (aOR1.88) and/or anosmia/ageusia (aOR 1.88) were significantly more likely to have SARS-CoV-2 infection. In addition, participants from Natitingou, those living in groups and even those with digestive symptoms were less likely to be infected. Among other things, we did not find a statistically significant association between age, sex, BMI and the existence of comorbidities (diabetes, hypertension, chronic lung disease) and test positivity.

Changes in national response strategies observed over the course of the study revealed that participants included in phase 3 of the study had a 3.16-fold higher risk of SARS-CoV-2 infection than those in previous phases (Table 3).

**Table 3:** Factors associated with the occurrence of SARS-CoV-2 infection in subjects volunteering for screening in Cotonou, Allada and Natitingou in 2021.

Factors	Number of participants infected with SARS-CoV2 (Prevalence [%])	Univariate analysis		Multivariate analysis^♣^	
		Crude odds-ratio [95% CI]	p-value	adjusted odds-ratio [95% CI]	p-value
Residence area	n = 4178				*<0.0001*
Cotonou	334 (15.27)	1		1	
Allada	172 (16.60)	1.10 [0.90–1.35]	0.330	2.04 [1.59–2.62]	<0.0001
Natitingou	42 (4.40)	0.25 [0.18–0.35]	<0.0001	0.27 [0.19–0.39]	<0.0001
Phase	n = 4178				*<0.0001*
Phase 1	116 (5.73)	1		1	
Phase 2	104 (10.79)	1.99 [1.51–2.63]	<0.0001	1.39 [0.99–1.94]	0.053
Phase 3	328 (27.61)	6.28 [5.01–7.88]	<0.0001	3.16 [2.34–4.27]	<0.0001
Gender	n = 4178			*	*
Male	265 (12.82)	1			
Female	283 (13.41)	1.05 [0.87–1.26]	0.575		
Age	n = 4178			*	*
[01 month–18 years]	38 (9.72)	1			
[18–35 years]	239 (13.26)	1.42 [0.99–2.04]	0.057		
[35–60 years]	225 (13.94)	1.50 [1.05–2.16]	0.027		
≥60 years	46 (12.40)	1.31 [0.83–2.07]	0.239		
BMI(kg/m^2^)	n = 3064			*	*
[18.5–25]	175 (11.51)	1			
<18.5	10 (5.32)	0.43 [0.22–0.83]	0.012		
[25–30]	147 (15.61)	1.42 [1.12–1.80]	0.004		
≥30	68 (16.43)	1.51 [1.11–2.05]	0.008		
Level of education	n = 4178				0.0046
Out of school	37 (6.39)	1		1	
Primary	72 (9.00)	1.45 [0.96–2.19]	0.078	1.25 [0.80–1.94]	0.319
Secondary	154 (12.61)	2.11 [1.46–3.07]	<0.0001	1.67 [1.12–2.51]	0.012
Higher	285 (18.06)	3.23 [2.26–4.61]	<0.0001	1.83 [1.22–2.74]	0.003
Profession	n = 4178			*	*
No professional activity	50 (8.18)	1	1		
Craftsman	33 (9.43)	1.17 [0.74–1.85]	0.509		
Shopkeeper/retailer	85 (16.87)	2.28 [1.57–3.30]	<0.0001		
Farmer	5 (2.16)	0.25 [0.10–0.63]	0.003		
School pupil/student	68 (12.66)	1.63 [1.11–2.39]	0.013		
Worker	19 (9.64)	1.20 [0.69–2.09]	0.524		
**Employee**	262 (17.07)	2.31 [1.68–3.17]	<0.0001		
Other	26 (12.21)	1.56 [0.94–2.58]	0.083		
Marital status	n = 3932			*	*
Single	172 (13.68)	1			
Married	345 (12.96)	0.93 [0.77–1.14]	0.492		
Type of habitat	n = 4178				*0.0207*
Individual	332 (12.15)	1			
Group	216 (14.95)	1.27 [1.06–1.53]	0.011	0.75 [0.60–0.94]	0.012
Diabetes	n = 4178				*0.9688*
No	534 (13.08)	1		1	
Yes	14 (14.89)	1.16 [0.65–2.07]	0.606	0.99 [0.52–1.86]	0.969
High blood pressure	n = 4178				*0.9501*
No	488 (13.08)	1		1	
Yes	60 (13.45)	1.03 [0.77–1.38]	0.824	0.99 [0.72–1.37]	0.955
Chronic lung disease	n = 4174				*0.1196*
No	483 (12.51)	1		1	
Yes	65 (20.77)	1.84 [1.37–2.45]	<0.0001	1.29 [0.94–1.79]	0.115
Self-medication with chloroquine	n = 2668			*	*
No	250 (20.83)	1			
Yes	218 (14.85)	0.66 [0.54–0.81]	<0.0001		
Functional symptoms	n = 4178				0.0183
Absent	109 (6.28)	1		1	
Present	439 (17.97)	3.27 [2.62–4.07]	<0.0001	1.35 [0.96–1.90]	0.083
General symptoms	n = 4178				0.0755
Absent	238 (8.83)	1		1	
Present	310 (20.92)	2.73 [2.28–3.28]	<0.0001	1.23 [0.97–1.57]	0.091
Respiratory symptoms	n = 4178				*<0.0001*
Absent	123 (6.61)	1		1	
Present	425 (18.33)	3.17 [2.57–3.92]	<0.0001	1.88 [1.40–2.53]	<0.0001
Digestive symptoms	n = 4178				0.0098
Absent	439 (12.83)	1		1	
Present	109 (14.42)	1.14 [0.91–1.44]	0.242	0.70 [0.54–0.91]	0.008
Anosmia/ageusia	n = 4178				*<0.0001*
Absent	352 (10.06)	1		1	
Present	196 (28.91)	3.64 [2.98–4.44]	<0.0001	1.88 [1.48–2.38]	<0.0001

♣ 4174 observations included in the final multivariate model.

* Variable removed from the final multivariate model.

Body mass index (BMI).

Variations in the factors associated with SARS-CoV-2 infection in the participants included were observed according to the study sites, the different changes in response strategy and the type of biological test (see Appendix).

## Discussion

**Fig 5:**
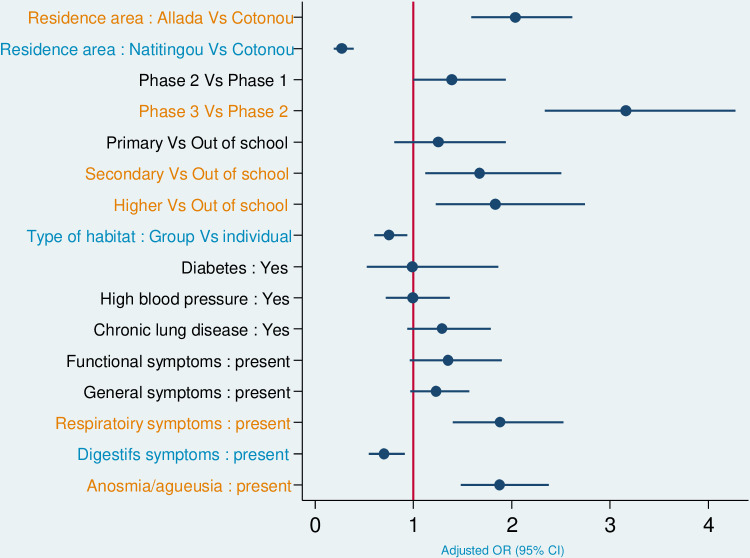
Multivariate logistic regression assessing the risk of SARS-CoV-2 infection in subjects volunteer for screening in Cotonou, Allada and Natitingou in 2021.

This study described the dynamics of SARS-CoV-2 infection and its factors at three sentinel sites in Benin (see Fig 5). The study period covered the end of the second wave (march-april) and the entire third wave of the COVID-19 epidemic in Benin (july-october 2021). During this study, a proportion of 13.1% of positive cases of SARS-CoV-2 infection was found over the surveillance. In 2021, Benin reported nationally around 4% of positive cases of SARS-CoV-2 infection [8]. The proportion of infected subjects observed in this study is consistent with the low rate of infected individuals reported nationally in Benin during the period [9], in other west African countries [2,24], or elsewhere such as in Zambia [24]. However, serological surveys in Africa have shown that these data do not reflect the reality of the spread of the epidemic, due to limited diagnostic resources and the very high proportion of asymptomatic or pauci-symptomatic cases who did not attend health facilities for screening [3–6].

The distribution of infected participants showed marked heterogeneity between the different study sites, with a higher proportion in Allada and Cotonou (in the south of the country, 17% and 15% respectively) than in Natitingou in the north (4%), as confirmed by multivariate analysis. This disparity can probably be explained in part by variations in population density between sites. Furthermore, as in many other countries around the world, the first cases of SARS-CoV-2 infection in Africa were associated with international contacts such as travel, trade, tourism and work-related travel [17], leading to more rapid spread in regions close to the initial entry points of the cases [11,13,17]. Cotonou, as Benin’s economic capital with an international airport, was a major gateway for SARS-CoV-2 cases from all over the world [17]. Less unexpectedly, the multivariate model revealed an excess risk in Allada, possibly due to this commune’s role as a commercial crossroads, attracting symptomatic individuals seeking screening, especially as it was the first site opened in Benin for the nationwide management of severe cases of COVID-19. In Natitingou, the low proportion of infected people was more predictable, given the relative isolation of this commune in northern Benin (some 500 km above and beyond the cordon sanitaire) [11]. National data corroborated the fact that the Atacora district (where Natitingou is located) had the lowest proportion of people infected with SARS-CoV-2 in Benin [8]. The geographical heterogeneity observed in the country [8,12,25] has also been observed, for example, in Mali, Sudan, Niger and Chad, with higher proportions of positive subjects in urban areas and relatively lower proportions in rural areas [17,26,27].

Over the course of the study, the patterns of positive cases observed (Fig 3) are in line with the national data [28], due to the different epidemic waves. In our data, gender was not associated with the risk of infection, in line with national statistics from Benin, but other studies have reported different results in Africa and worldwide, with an increased risk of SARS-CoV-2 infection in men [26,27,29].

Our results also show that people living in larger households are less likely to be infected with SARS-CoV-2. Another study conducted before (and during) the adoption of containment measures in UK did not find this association [15]. However, our study was carried out in a very different epidemiological context, it is therefore difficult to compare with our results. This seems to suggest that intra-family contamination was probably not the highest risk of infection in Benin.

In addition, participants with secondary or higher education had a risk of infection 1.67 to 1.83 times higher than those with no education. On the other hand, a weak association was shown in a previous study between less educated people and SARS-CoV-2 infection [13]. It should be noted that in our sample, there was an over-representation of educated people, with 67% of participants having a secondary or higher level of education, whereas in 2018, the fifth demographic and health survey in Benin indicated that around 30% of Beninese were in these groups [30]. This over-representation suggests that more educated people are more likely to come for spontaneous testing. One possible hypothesis is that more educated people are more aware of the risk of SARS-CoV-2 infection and are therefore more likely to come forward for testing. They may also be more likely to experience symptoms.

Numerous studies have highlighted the link between the existence of co-morbidities such as obesity, diabetes mellitus, hypertension and chronic lung disease, and the severe development and mortality associated with COVID-19 [31–35]. Some research has also suggested a possible association between certain co-morbidities, such as acute renal failure, and SARS-CoV-2 infection [12,15]. However, our study found no association between these comorbidities and COVID-19 test positivity in any of the participants. Age, on the other hand, has been identified in the literature as a factor associated with SARS-CoV-2 infection [15], as well as severe forms of COVID-19, with an increased risk in individuals aged 40 and over, characterised by high viral load and admissions to intensive care [13,16]. Despite the considerable contribution of these two factors to the spread of the disease in Europe and America [15], Africa has experienced reduced transmission of COVID-19 due to its predominantly young population and low prevalence of co-morbidities [3,15,24]. Our results are consistent with these observations. These elements constitute solid arguments explaining the differences in results observed between Africa and Western countries.

Respiratory symptoms and/or anosmia/ageusia were also associated with SARS-CoV-2 infection. Symptoms such as fever, cough, sputum and dyspnoea occurring within the first few days of infection have often been reported [36–38], and anosmia and ageusia are signs of olfactory impairment which, associated with a respiratory syndrome, have been described as more specific symptoms of SARS-CoV-2 infection [39,40], in agreement with our results. Although rare according to the literature [32,35,41], digestive symptoms were observed in some study participants, revealing them to be less likely to have SARS-CoV-2 infection. It should be emphasized that, although present, these symptoms are not specific to the clinical picture of pulmonary virosis typically described in the case of SARS-CoV-2 infection.

These results are of interest in terms of public health because they show the heterogeneity of the risk of infection within the population. Knowing this heterogeneity during an epidemic is important for identifying groups at risk of infection and directing the response, in particular by targeting awareness and prevention campaigns at these groups.

This study was carried out in centres involved in the management of cases of SARS-CoV-2 infection in the three study districts. One of the strengths of this study was that it set up sentinel surveillance at various sites, enabled the centres’ staff to be strengthened to better meet demand, collected comprehensive data that was richer than the routine questionnaires, and ensured a rapid reporting system for the results and sophisticated statistical analysis. All this enabled us to identify geographical heterogeneity and risk factors for SARS-CoV-2 infection in the study sites.

Nevertheless, our results cannot be extrapolated to a national health situation since the geographical heterogeneity observed in our study is probably not representative of the overall heterogeneity around the country. Another limitation of the study was that a selection bias was admitted, as the subjects included in the study decided on their own to be screened. This led, for example, to an over-representation of people with higher education compared to the general population. This project actually took place in an epidemic emergency context constantly evolving with in particular diagnostic tools that have evolved during the health crisis. Unlike a traditional research project, it was therefore necessary to adapt to this evolution. Due to the evolution of national COVID-19 response strategies, the project went through different phases and two different biological tests were used during the study (RT-PCR and COVID-19 antigenic RDTs) which had different sensitivities, making our results more complex to interpret. However, we globally obtained interesting and consistent results, and we carried out additional analyses (see appendix) which allow us to be confident in the conclusions reached here.

## Conclusion

This work is in favour of encouraging the rapid implementation of sentinel surveillance at several national sites spread over different representative areas of the country (urban, semi-urban, rural) with COVID-19 diagnostic capabilities (PCR). This, combined with real-time data analysis, would enable to better characterise the spread of the epidemic and the most at risk population groups. However, in the case of an infection such as SARS-COV-2, which results in a large proportion of asymptomatic or pauci-symptomatic individuals, this antigenic surveillance must be combined with serological surveillance to better estimate the actual proportion of people who have been in contact with the virus, in order to give a more accurate picture of the extent of the spread of the virus in the population and across the country.

## Appendices

Changes in the detection strategy for COVID-19-positive cases revealed that factors associated with SARS-CoV-2 infection varied according to the type of bioassay used. In Allada, participants were 6 times more likely to be infected with SARS-CoV-2 when an antigenic RDT was used, compared with 4 times more likely when RT-PCR was used. In Natitingou, susceptibility to infection was halved when antigenic RDT was used, versus 66% when RT-PCR was used. The presence of functional symptoms and/or anosmia/ageusia was twice as likely with a positive antigenic RDT. However, when RT-PCR was used, the presence of respiratory symptoms and/or anosmia/ageusia was three times more likely in the case of SARS-CoV-2 infection, compared with a halving in the presence of digestive symptoms (Table 4).

**Table 4:** Effects of modifying biological tests for SARS-CoV-2 infection.

	PCR sympto (n = 1184)		TDR sympto (n = 968)	
	aOR_1_ [95% CI_1_]	p value	aOR_2_ [95% CI_2_]	p value
**Residence area**				
Cotonou	1		1	
Allada	4.22 [2.77–6.42]	0.000	5.51 [2.39–12.73]	0.000
Natitingou	0.14 [0.06–0.35]	0.000	0.49 [0.29–0.81]	0.006
**Level of education**				
Out of school	1		1	
Primary	3.43 [1.44–8.17]	0.005	1.49 [0.58–3.82]	0.405
Secondary	5.26 [2.32–11.88]	0.000	1.55 [0.64–3.76]	0.335
Higher	5.32 [2.35–12.07]	0.000	1.77 [0.74–4.24]	0.200
**Type of habitat**				
Individual	1		1	
Group	0.72 [0.47–1.08]	0.117	0.72 [0.51–1.02]	0.071
**Diabetes**				
No	1		1	
Yes	1.86 [0.72–4.80]	0.199	0.96 [0.36–2.56]	0.940
**High blood pressure**				
No	1		1	
Yes	0.92 [0.55–1.55]	0.761	0.90 [0.53–1.52]	0.699
**Chronic lung disease**				
No	1		1	
Yes	1.68 [0.90–3.15]	0.104	1.36 [0.90–2.06]	0.149
**Functional symptoms**				
Absent	1		1	
Present	1.07 [0.60–1.90]	0.815	2.24 [1.10–4.58]	0.026
**General symptoms**				
Absent	1		1	
Present	1.07 [0.73–1.58]	0.707	1.43 [0.98–2.08]	0.064
**Respiratory symptoms**				
Absent	1		1	
Present	3.00 [1.80–4.98]	0.000	1.07 [0.64–1.78]	0.795
**Digestive symptoms**				
Absent				
Present	0.56 [0.34–0.92]	0.023	0.78 [0.55–1.09]	0.148
**Anosmia/agueusia**				
Absent	1		1	
Present	2.93 [1.92–4.45]	0.000	1.46 [1.06–2.03]	0.021

aOR, adjusted odds-ratio, 95% CI_x_, 95% confidence interval

Participants’ clinical status, whether symptomatic or asymptomatic, also influenced factors associated with SARS-CoV-2 infection. In Natitingou, participants were less likely to be infected when they were all screened, regardless of their clinical status. This susceptibility was even lower when only symptomatic subjects were screened. The presence of respiratory signs and/or anosmia/ageusia was three times more likely in the case of a positive COVID-19 test when all subjects were symptomatic, and less than twice as likely when all subjects were screened (Table 5).

**Table 5:** Effects of changing the target population for screening for SARS-CoV-2 infection.

	PCR sympto & asympto (n = 2022)		PCR sympto (n = 1184)	
	aOR_1_ [95% CI_1_]	p value	aOR_2_ [95% CI_2_}	p value
**Residence area**				
Cotonou	1		1	
Allada	1.15 [0.74–1.77]	0.534	4.22 [2.77–6.42]	0.000
Natitingou	0.18 [0.08–0.42]	0.000	0.14 [0.06–0.35]	0.000
**Level of education**				
Out of school	1		1	
Primary	0.67 [0.34–1.33]	0.256	3.43 [1.44–8.17]	0.005
Secondary	1.11 [0.62–1.98]	0.728	5.26 [2.32–11.88]	0.000
Higher	1.33 [0.73–2.41]	0.353	5.32 [2.35–12.07]	0.000
**Type of habitat**				
Individual	1		1	
Group	1.12 [0.75–1.68]	0.573	0.72 [0.47–1.08]	0.117
**Diabetes**				
No	–		1	
Yes	–		1.86 [0.72–4.80]	0.199
**High blood pressure**				
No	1		1	
Yes	1.43 [0.71–2.86]	0.315	0.92 [0.55–1.55]	0.761
**Chronic lung disease**				
No	1		1	
Yes	0.77 [0.31–1.90]	0.578	1.68 [0.90–3.15]	0.104
**Functional symptoms**				
Absent	1		1	
Present	0.85 [0.43–1.65]	0.627	1.07 [0.60–1.90]	0.815
**General symptoms**				
Absent	1		1	
Present	1.91 [1.04–3.50]	0.036	1.07 [0.73–1.58]	0.707
**Respiratory symptoms**				
Absent	1		1	
Present	2.10 [1.20–3.68]	0.009	3.00 [1.80–4.98]	0.000
**Digestive symptoms**				
Absent	1		1	
Present	1.21 [0.56–2.61]	0.632	0.56 [0.34–0.92]	0.023
**Anosmia/agueusia**				
Absent	1		1	
Present	2.10 [1.06–4.18]	0.034	2.93 [1.92–4.45]	0.000

aOR: adjusted odds-ratio; 95% CI_x_: 95% confidence interval

Level of education appeared to be increasingly supportive of SARS-CoV-2 infection as one progressed from primary to tertiary level, but only in symptomatic subjects screened by PCR.

## Supporting information

S1 File STROBE checklist. (DOCX)
